# Frequency of intravenous therapy use in elite adult track and field athletes

**DOI:** 10.3389/fspor.2026.1810913

**Published:** 2026-06-22

**Authors:** Eduard Bezuglov, Evgeny Achkasov, Ryland Morgans, Artemii Lazarev, Oleg Talibov, Elizaveta Kapralova, Elizaveta Koneva, Georgiy Malyakin

**Affiliations:** 1 Department of Sports Medicine and Medical Rehabilitation, Sechenov First Moscow State Medical University, Moscow, Russia; 2 Northwest Health, Chesterton, Chicago, United States; 3 Department of Therapy, Clinical Pharmacology and Ambulance Care, Russian University of Medicine, Moscow, Russia

**Keywords:** elite athletes, infusions, intravenous drip, intravenous fluids, parenteral

## Abstract

**Objective:**

To investigate the prevalence, patterns and substances of intravenous (IV) therapy among elite endurance athletes.

**Design:**

Cross-sectional observational study

**Methods:**

Participants were anonymously surveyed through a specially designed questionnaire. Responses to the questionnaire were received between October 2020 and January 2021. IV use of various medications and its association with sex, age, sporting discipline and competition tier was assessed.

**Results:**

Among 138 elite track and field athletes (73 men and 65 women) 56.5% (*n* = 78) reported IV administration of various drugs not prohibited by the World Anti-Doping Agency. Within this group, 18.8% (*n* = 26) administered these drugs regularly and 37.7% (*n* = 52) during the preparation for major competitions only competition. Actovegin (77.4%), phosphocreatine (64.5%), amino acids (58.1%), essentiale (57.0%), heptral (47.3%) and reamberin (44.1%) were most commonly used for this purpose. Comparison by competition level criterion revealed more frequent use of IV therapy by international level athletes compared to national level athletes (*p* = 0.02, OR 2.43 1.14–5.15). In addition, extra-level athletes were also significantly more likely to use amino acids (*p* = 0.016) and essentiale (*p* = 0.024) compared to national-level athletes. There were no statistically significant differences in the prevalence of parenteral medication use between international and extra-level athletes.

**Conclusions:**

The use of IV therapy among elite track and field athletes appears to be relatively common based on self-reported data.

## Introduction

Modern elite sport places high demands on the human body. Athletes often use various substances to improve physical performance and recovery to achieve the highest level of success. However, a number of studies indicate that athletes are much more likely to use prohibited substances and methods than official statistics suggest (1–3). Most athletes will not intentionally use prohibited substances and methods, but there will often be a desire to maximise performance through the use of legal ergogenic aids (4–6). It should be noted that all intravenous (IV) infusions of more than 100 millilitres in an interval of less than 12-hours are currently banned by the World Anti-Doping Agency (WADA) (7). At the same time, there is evidence that 75% of National Football League athletes use pre-game IV fluids. This may be due to the lack of anti-doping restrictions on the use of IV infusions in this league (8). Faced with high intensity workloads and busy competitive and training schedules athletes and staff try to optimise recovery processes. In this case, IV therapy as a rapid method of rehydration and delivery of ergogenic substances may be employed.

To date, there are no high methodological studies that have investigated the potential benefits of IV administration of ergogenic substances compared to the standard oral intake for athletes. However, it is no secret to practitioners that some healthy athletes regularly use IV nutritional products. Athletes intravenously administer electrolytes, amino acids, vitamins B and C, glutathione and iron. However, this data was not obtained as a result of scientific research involving athletes but rather represents the personal opinion of certain specialists, passed along through word of mouth (9).

The scarcity of information regarding the prevalence of IV therapy use by elite athletes and the drugs administered makes it difficult to truly assess the prevalence of this phenomenon in professional sport or its efficacy compared to the oral form. Thus, the purpose of this study is to investigate the prevalence, patterns and substances used during IV therapy among elite endurance athletes.

## Methods

### Design

Cross-sectional observational study (level of evidence III).

### Subjects

A total of 138 Russian adult track and field athletes that had performed or continue to perform at the top tier submitted responses. Of the participants, 52.9% were men (*n* = 73, age 24.0 ± 4.4, weight 65.0 ± 6.0 kg, height 178 ± 6.0 cm), and 47.1% were women (*n* = 65, age 22.8 ± 3.8, weight 50.4 ± 5.0 kg, height 165 ± 5.5 cm). The responses to questionnaire were submitted by athletes competing in racewalking and middle- and long-distance running (distances of 800–5,000 m and 10,000 m or longer, respectively). Overall, 66 athletes were participants of national track and field championships (group “national level”) and 60 athletes had participated in major international tournaments, i.e., Olympic Games, World and European Championships (group “international level”). Additionally 12 participants had received awards in major international competitions (group “extra tier”) (Table 1). The “extra tier” subdivision is due to the fact that this group of athletes receives substantial financial support in Russia, which provides them with greater access to various medications and medical care.

**Table 1:** Demographic and competitive characteristics of the study participants by tier.

Athletes's group	National tier *n* (%)	International tier *n* (%)	Extra tier *n* (%)	Overall
Men	40 (54.8)	27 (37.0)	6 (8.2)	73
Women	26 (40.0)	33 (50.8)	6 (9.2)	65
Racewalking	21 (31.8)	35 (53.1)	10 (15.1)	66
Middle-distance running	27 (64.3)	13 (30.9)	2 (4.8)	42
Long-distance running	18 (60.0)	12 (40.0)	0 (0.0)	30

The study was performed in accordance with the Declaration of Helsinki and was approved by the local ethics committee (N 05-21 dated 10.03.2021). Informed consent was obtained from all participants.

### Inclusion and exclusion criteria

Inclusion criteria: written consent to participate in the study; age 18 years and older; participation in the national athletics teams; no previous disqualifications for anti-doping rule violation or other reasons.

Exclusion criteria: Current use of medication for the treatment of any diagnosed medical condition; psychosocial or emotional conditions; or any cardiovascular conditions within the past two years, that could influence the pharmacodynamics or pharmacokinetics of intravenous medications.

### Patient and public involvement statement

Athletes were not involved in setting the research question or the outcome measures, but were intimately involved in design as they added more options in one of the questions. Participants were important to dissemination of the baseline information, which helped to motivate community involvement during and beyond the study.

### Equity, diversity, and inclusion statement

The research team included 7 men and 2 women from different disciplines (clinical medicine, rehabilitation, sports coaching, epidemiology), including three authors considered junior scholars. The study population included a spectrum of ages, sexes, demographics and competitive levels. Our analysis explores the effects of sex and competitive level; however, we acknowledge we did not examine the effects of race and ethnicity.

### Procedures

To analyse the IV therapy medications used by highly competitive endurance athletes in preparation for important sporting events, the athletes were invited to complete an online questionnaire, which was created using Google Forms. The questionnaire was designed by the current authors (Table 2). In addition, respondents were asked to rank the effectiveness of the IV medications used. In a pre-test, the questions were presented to a group of 10 semi-professional track and field athletes. These answers were not considered in the analysis.

**Table 2:** The questionnaire “Use of intravenous therapy among highly competitive endurance athletes”.

Sex	
□ Men	
□ Women	
Main discipline:	Qualification
□ Middle-distance running (800–5,000 m)	□ National Tier
□ Distance run (10,000 m or above)	□ International Tier
□ Race walking	□ Extra Tier
How often do you use intravenous therapy? If the answer to the previous question is no, why?	
□ Yes, regularly during the training process	
□ Yes, during the preparation for major competitions only	
□ No, I think it was not necessary	
□ No, I did not have the opportunity	
□ No, I was afraid of breaking anti-doping rules	
What intravenous medications did you use? (Multiple options can be selected)	
□ Phosphocreatine	□ L-Carnitine
□ Actovegin	□ Esafosfina
□ Reamberin	□ Heptral
□ Cytoflavin	□ Essentiale
□ Amino acids	□ TAD (Glutathione)
□ Other: __________	
What intravenous medication mentioned above do you consider the most effective? Name the five in descending order of effectiveness: ___________________________________________________________________________	

Responses to the questionnaire were received between October 2020 and January 2021. IV use of various medications and its association with sex, age, sporting discipline and competition tier was assessed. No personally identifiable information (such as names, email addresses, or contact details) was collected. Settings were configured to avoid automatic collection of identifying metadata where possible, and responses were stored in a secure, password-protected database accessible only to the research team.

Prior to participation, all respondents were presented with an electronic participant information sheet outlining the study purpose, procedures, voluntary nature of participation, and data confidentiality. Participants were required to provide informed consent by actively selecting an agreement option before accessing the questionnaire. Only participants who provided consent were able to proceed. Submission of the completed questionnaire was considered as documentation of informed consent.

### Bias

Given the sensitive nature of intravenous therapy use in a high-performance sport and anti-doping context, several measures were implemented to reduce the risk of response bias, including social desirability bias and underreporting (10). The questionnaire was administered anonymously, and no personally identifiable information was collected, thereby minimizing concerns about potential consequences of disclosure. In addition, the survey was intentionally limited to questions regarding intravenous therapies within permitted volumes and commonly used legal substances by WADA, avoiding direct inquiry into prohibited practices. Participants were informed that responses would be analyzed in aggregated form only (11).

### Statistical analysis

The data was processed in Microsoft Excel and analysed using GraphPad Prism 9 (GraphPad Software, San Diego, CA, USA). Kolmogorov–Smirnov test was used to assess the normality of the distribution. Mann–Whitney U-test (or Student's t-test for normally distributed data) was used to analyse the relationship between pairs of numerical values. Although the data were assessed for normality, the majority of variables did not meet normality assumptions; therefore, non-parametric approaches were applied. Consequently, ANOVA and related *post-hoc* tests were not used. Instead, group comparisons were performed using appropriate non-parametric or categorical tests (Chi-squared test and Fisher's *F*-test), and effect sizes were expressed as odds ratios with 95% confidence intervals. Significance was set at *p* < 0.05.

## Results

Data was provided by 132 of the 138 survey participants. Nearly 57% of athletes confirmed the use of IV administration of various medications not prohibited by WADA. Responses to both the frequency of IV therapy use in general and the use of specific medications are provided (Tables 3, 4). Each athlete who used IV therapy entered one or more medications into the questionnaire.

**Table 3:** Prevalence of intravenous administration of non-prohibited substances.

Frequency of use	Men *n* (%)	Women *n* (%)	Total *n* (%)
Regularly during the training process	12 (16.4)	14 (21.5)	26 (18.8)
During the preparation for major competitions only	26 (35.7)	26 (40.1)	52 (37.7)
No, I think it was not necessary	12 (16.4)	6 (9.2)	18 (13.0)
I did not have the opportunity	8 (11.0)	4 (6.1)	12 (8.7)
I was afraid of breaking anti-doping rules	12 (16.4)	12 (18.5)	24 (17.4)
No response	3 (4.1)	3 (4.6)	6 (4.3)

**Table 4:** List of medications administered intravenously among participants who reported the substances used.

Substance	Men (*n* = 53) *n* (%)	Women (*n* = 40) *n* (%)	Total (*n* = 93) *n* (%)
Phosphocreatine	27 (50.9)	33 (82.5)	60 (64.5)
Actovegin	33 (62.3)	39 (97.5)	72 (77.4)
Reamberin	22 (41.5)	19 (47.5)	41 (44.1)
Cytoflavin	17 (32.1)	13 (32.5)	30 (32.2)
Amino acids	26 (49.1)	28 (70.0)	54 (58.1)
L-Carnitine	21 (39.6)	16 (40.0)	37 (39.8)
Esafosfina	14 (26.4)	8 (20.0)	22 (23.6)
Heptral	24 (45.3)	20 (50.0)	44 (47.3)
Essentiale	24 (45.3)	29 (72.5)	53 (57.0)
TAD (glutathione)	15 (28.3)	10 (25.0)	24 (25.9)

Phosphocreatine was viewed as the most effective parenteral medication among both men and women participants. It was ranked first by 17 men and 26 women. Moreover, phosphocreatine (23 times), essentiale (15 times), actovegin (13 times), Glutathione (TAD) (13 times), heptral (13 times), esafosfina (13 times) were listed by men as the five most effective medications. Among women, the top five preferences were phosphocreatine (36 times), actovegin (30 times), amino acids (28 times), essentiale (21 times), heptral (20 times) (Table 5).

**Table 5:** Weighted effectiveness scores of intravenously administered substances among participants who specified their preferred substances (*n* = 72).

Substance	Men score	Women score	Total score
Phosphocreatine	99	163	262
Actovegin	44	90	134
Amino acids	38	83	121
Essentiale	40	50	90
Heptral	32	57	89
Esafosfina	43	40	83
TAD (Glutathione)	44	32	76
Reamberin	30	35	65
Cytoflavin	22	15	37
L-Carnitine	8	29	37

There was no statistically significant difference in the use of IV therapy overall (*p* = 0.23) and for specific drugs separately when analysing sex differences.

No statistically significant difference was found in IV therapy use overall (*p* = 0.46) when analysed by athlete age. However, the group of athletes consuming IV amino acids was significantly older than the group not using the supplement (*p* = 0.011).

Comparison by competition level criterion revealed more frequent use of IV therapy by international-level athletes compared to national-level athletes (*p* = 0.02, OR = 2.43, 95% CI 1.14–5.15). International-level athletes were significantly more likely to use essentiale (*p* < 0.001, OR = 5.2, 95% CI = 2.4–11.8) and phosphocreatine (*p* = 0.001, OR = 3.4, 95% CI = 1.6–1.1) than national-level athletes. In addition, extra-level athletes were also significantly more likely to use amino acids (*p* = 0.016, OR = 4.54, 95% CI = 0.06–0.81) and essentiale (*p* = 0.024, OR = 4.16, 95% CI = 0.07–0.89) compared to national-level athletes. There were no statistically significant differences in the prevalence of parenteral medication use between international- and extra-level athletes.

No statistically significant differences were found between the three disciplines studied (*p* = 0.15).

## Discussion

The results of this study demonstrated that more than 50% of elite Russian track and field athletes used IV administration of various substances during their career. Consequently, the prevalence of IV usage is notably high (> 50%) among elite track and field athletes. However, IV therapy was not administered for rapid rehydration (in the case of severe dehydration) or for iron deficiencies, which are the only relative indications for the use of IV therapy in sport. It is important to highlight that in the case of IV rehydration obtaining a Therapeutic Use Exemption (TUE) is required, as volumes exceeding 100 mL are used (12–16).

More than 75% of elite track and field athletes considered the use of IV therapy to be beneficial for sporting success. Furthermore, 22% of participants did not use this method of administration solely due to potentially violating anti-doping rules or did not have such an opportunity.

This study may be the first to assess the prevalence and composition of IV therapy employed by elite track and field athletes and this may be the highest prevalence recorded.

When analysing the substances most frequently used by these track and field athletes, the most commonly administered were actovegin (77.4%), phosphocreatine (64.5%), amino acids (58.1%), essentiale (57.0%), heptral (47.3%) and reamberin (44.1%). Among these substances, only phosphocreatine has evidence of a positive effect on performance. However, athletes of both sexes identified phosphocreatine as the most effective for recovery. It is clear that the protocol for IV phosphocreatine cannot be similar to the most common protocol of oral intake in sport (0.3 grams per kg/body weight in the loading phase followed by 0.03 grams per kg/body during the maintenance phase) (17). However, no studies have demonstrated superior efficacy of intravenous phosphocreatine compared to oral administration, especially given the high bioavailability associated with the oral route (18, 19).

The use of amino acids to optimise recovery should not be surprising, however, given the volume of amino acid solution required by elite athletes during the most intense periods of training, this protocol is not logical. Obviously, supplementing amino acids orally is more convenient, safer, and effective (20).

Another frequently employed substance is reamberin, which is a solution consisting of a mixture of various electrolytes and succinic acid. According to the manufacturer's statement, it has an antihypoxic and antioxidant effect, positively influencing aerobic processes in the cell, reducing the production of free radicals and restoring the energy potential of cells (21). This drug, along with hypoxen and previously meldonium, is considered as one of the most commonly used substances by athletes from one geographical region, the former Soviet Union. However, there are no studies of high methodological quality assessing its effectiveness in optimising recovery. Furthermore, the oral intake of electrolytes and succinic acid by healthy athletes allows any deficiency to be replenished without resorting to venous access.

It should be noted that the list of frequently used substances for IV administration from the examined participants also includes cytoflavin, another drug, along with reamberin, which is produced only in Russia and which includes inosine, nicotinamide, riboflavin and succinic acid.

Actovegin is the most commonly used substance in athletes of both sexes and there is still a lack of research on its effects on exercise performance and recovery. Actovegin is a purified deproteinised haemodialysate extracted from bovine blood by ultrafiltration. It consists of electrolytes, micronutrients, a combination of amino acids and intermediate products of carbohydrate metabolism. The mechanism of its action on muscle tissue repair is not sufficiently studied, and the data obtained are contradictory. There is some evidence of its positive effect on stimulation of myoblasts and enhancement of their fusion, as well as enhancement of oxidative function of mitochondria (22, 23). The aforementioned data may suggest a rationale for its use as a recovery aid, particularly given the absence of oral forms.

Some substances reported by participants are used quite frequently by athletes from other countries according to unpublished data: TAD (glutathione), esafosfina, and L-carnitine. These were rarely used by the study group, although, for example, esafosfina has been rated among the most effective by male track and field athletes. Most likely, one of the important reasons for the rare use of these substances could be the high cost and the unavailability of official purchase for the examined participants.

Heptral and Essentiale were among the most frequently used IV substances reported by the athletes, although the evidence supporting their use for performance recovery remains limited. Heptral is generally considered to have a stronger choleretic and metabolic effect, and it has shown potential benefit in some settings of liver dysfunction and cholestasis (24, 25). In contrast, Essentiale is primarily used as a phospholipid-based, membrane-protective agent with supportive effects on hepatic cell repair (26). In the context of sport, however, neither drug has strong high-quality evidence demonstrating a clear advantage for recovery or performance enhancement in healthy athletes. Their popularity appears to reflect a belief in hepatoprotective and restorative effects rather than a well-established ergogenic rationale, which makes their routine intravenous use difficult to justify outside specific medical indications.

When assessing the prevalence of IV administration of various substances, it is worth mentioning anti-doping legislation and the potential complications associated with this intervention. According to manufacturers’ instructions and private information from track and field coaches, the protocol for the use of the substances mentioned in this study allows for intravenous administration without violating anti-doping rules, i.e., no more than 100 mL at intervals of at least 12 h. However, the risk of violating these rules when performing intravenous injections can still be described as high.

### Clinical implications

The findings may help optimise the administration routes of various substances among elite endurance athletes. Additionally, the data could assist anti-doping organizations in estimating the actual prevalence of intravenous infusions of approved substances that may exceed permissible limits.

Intravenous infusions require special equipment and trained personnel and can lead to a variety of complications. Although precise data on the number of complications are currently unavailable, data on the use of IV catheters are accessible. The most common complications include thrombophlebitis (up to 44% with catheter use), bleeding, hematoma, soft tissue infiltration (16.3%), and less commonly syncope and air embolism (27–31). However, neither the availability of oral analogues, the lack of rigorous evidence of efficacy, the relatively high risk of complications, nor the high risk of anti-doping violations is preventing high-level track and field athletes from using intravenous therapy. This may be related to the belief in the stronger effect and full absorption of the substances in the IV route of administration, as well as the apparently lower risk of contamination with prohibited substances, as it has been shown that contamination of dietary supplements can exceed 50% (32–34). In this case, the likelihood of contamination of infusion solutions is reduced due to stringent manufacturing requirements.

At the same time, the most effective method of reducing the use of IV therapy can only be a complete ban by the WADA, with an exception for life-threating conditions (allergic reactions, surgical procedures, bleeding, severe anaemia and severe dehydration) and where retrospective therapeutic use exemptions are possible.

Future research should focus on incorporating IV therapy substances, such as phosphocreatine, as control variables in longitudinal studies to measure their direct effects on performance and recovery.

### Limitations

The limitations of this study include the cross-sectional method used for this work. Furthermore, the research was conducted during COVID outbreak. Another limitation was the absence of inquiries regarding the effects of these substances on athletic performance. The small sample is also a limitation of this study, although the small pool of potential participants contributes to this limitation. Finally, this study is focusing solely on racewalking and middle- to long-distance running, which represents a limited subset of track and field athletes. The measurement of IV therapy “frequency” is not clearly operationalised. Categories such as “regularly” or “during preparation for major competitions” are subjective and lack temporal definition, limiting interpretability. The study therefore supports a binary classification of use versus non-use, rather than meaningful frequency or behavioural pattern analysis.

Further studies should investigate both the efficacy of these substances on physical performance and recovery, and conduct comparative analyses of their efficacy across different routes of administration. Additionally, future research should expand the scope to include participants from high intensity running events, such as sprinting and jumping, as well as non-running disciplines like throwing.

## Conclusions

The use of IV therapy among elite track and field athletes appears to be relatively common based on self-reported data. The substances most frequently reported are consistent with those permitted under current anti-doping regulations when administered within established limits. The possible risk of complications and the lack of evidence regarding the efficacy of enhancing performance and accelerating recovery of the most commonly used substances in track and field must be considered.

## Data Availability

The raw data supporting the conclusions of this article will be made available by the authors, without undue reservation.
